# Supplementary figures and images for: Biochemical analysis to study wild-type and polyglutamine-expanded ATXN3 species
Source: PLoS One. 2024 Dec 23;19(12):e0315868. doi: 10.1371/journal.pone.0315868 (PMC11666052; doi:10.1371/journal.pone.0315868)

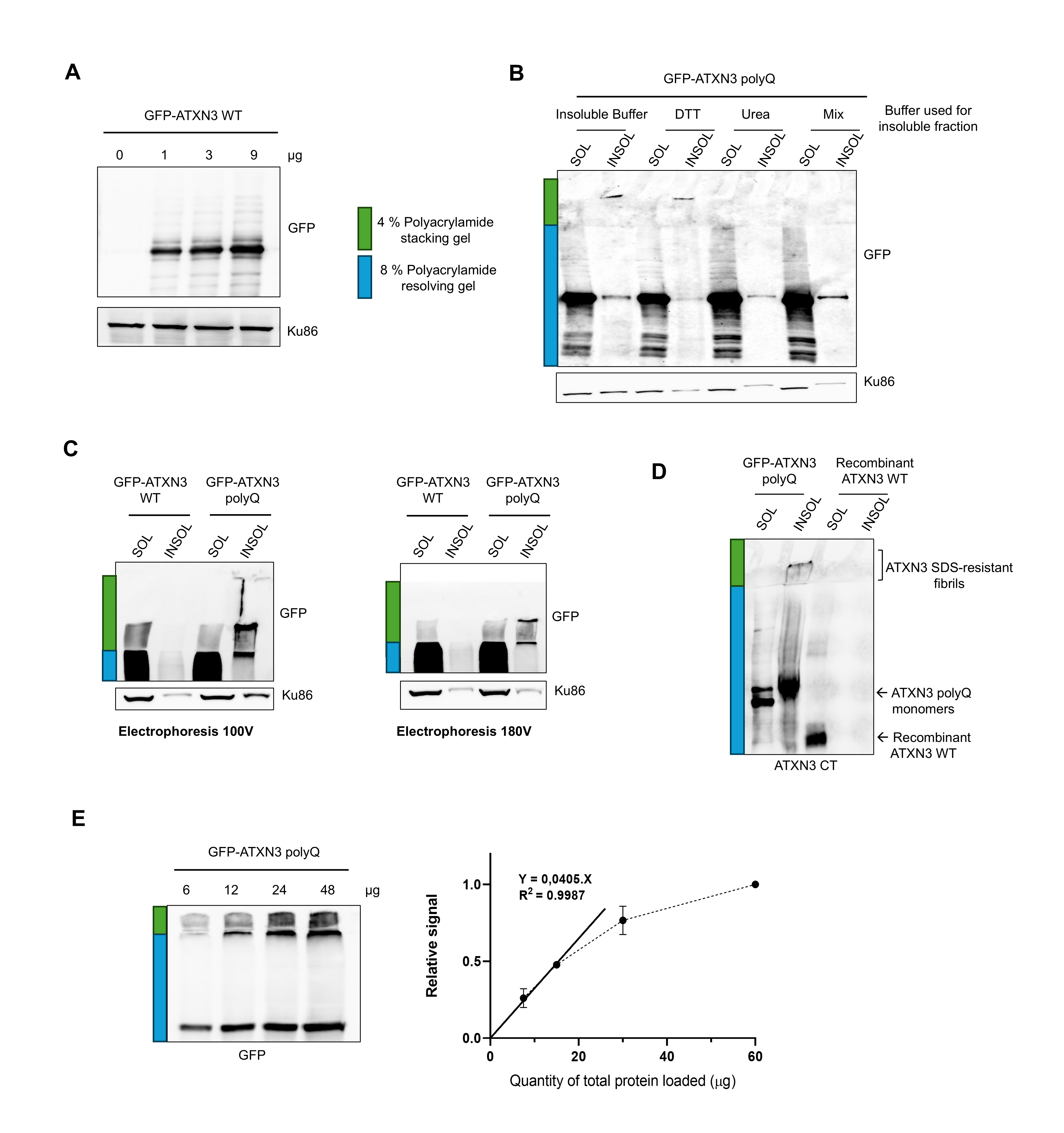

Supplement: S1 Fig — (A) HEK-239T were transfected with the indicated plasmid amounts to express GFP-ATXN3 WT. After 48h, cells were lysed and total cell extracts were analysed by SDS-PAGE with the indicated antibody. (B) HEK-239T were transfected with the GFP-ATXN3 polyQ-expressing plasmid and collected 7 days post-transfection. Cells were lysed and fractionated as described in the methods section, except for the last step. After the last wash and centrifugation, the insoluble pellet was resuspended in various buffers: the insoluble buffer containing 4% SDS, a DTT buffer (50mM Tris 7.4, 150 mM NaCl, 100 mM DTT), a Urea buffer (30 mM Tris pH 8.5, 7 M urea, 2 M thiourea) and a mix buffer (30 mM Tris pH 8.5, 7 M urea, 2 M thiourea, 100 mM DTT, 4% SDS). Subsequently, soluble and insoluble fractions were analysed by SDS-PAGE and membranes were immunoblotted for GFP. (C) HEK-239T transfected with GFP-ATXN3 polyQ, were lysed 7 days after transfection, fractionated and run on SDS-PAGE before being immunoblotted with the indicated antibodies. Electrophoresis was performed with 2 different voltages. (D) Purified His-tagged ATXN3-WT protein and extracts from cells expressing GFP-ATXN3 polyQ for 7 days were fractionated using the protocol described in the methods. Soluble and insoluble fractions were analysed by western blot with an antibody raised against the C-terminus of ATXN3 (E) Increasing amounts of insoluble fractions of cells expressing GFP-ATXN3 polyQ for 7 days were analysed by SDS-PAGE. The GFP signal from the stacking gel of 3 replicates was quantified, and a linear correlation was applied between the quantity of protein loaded and the GFP signal. The GFP signal in the stacking gel was measured using ImageJ and quantifications were plotted against the amount of protein loaded. (TIF) [file pone.0315868.s001.tif]

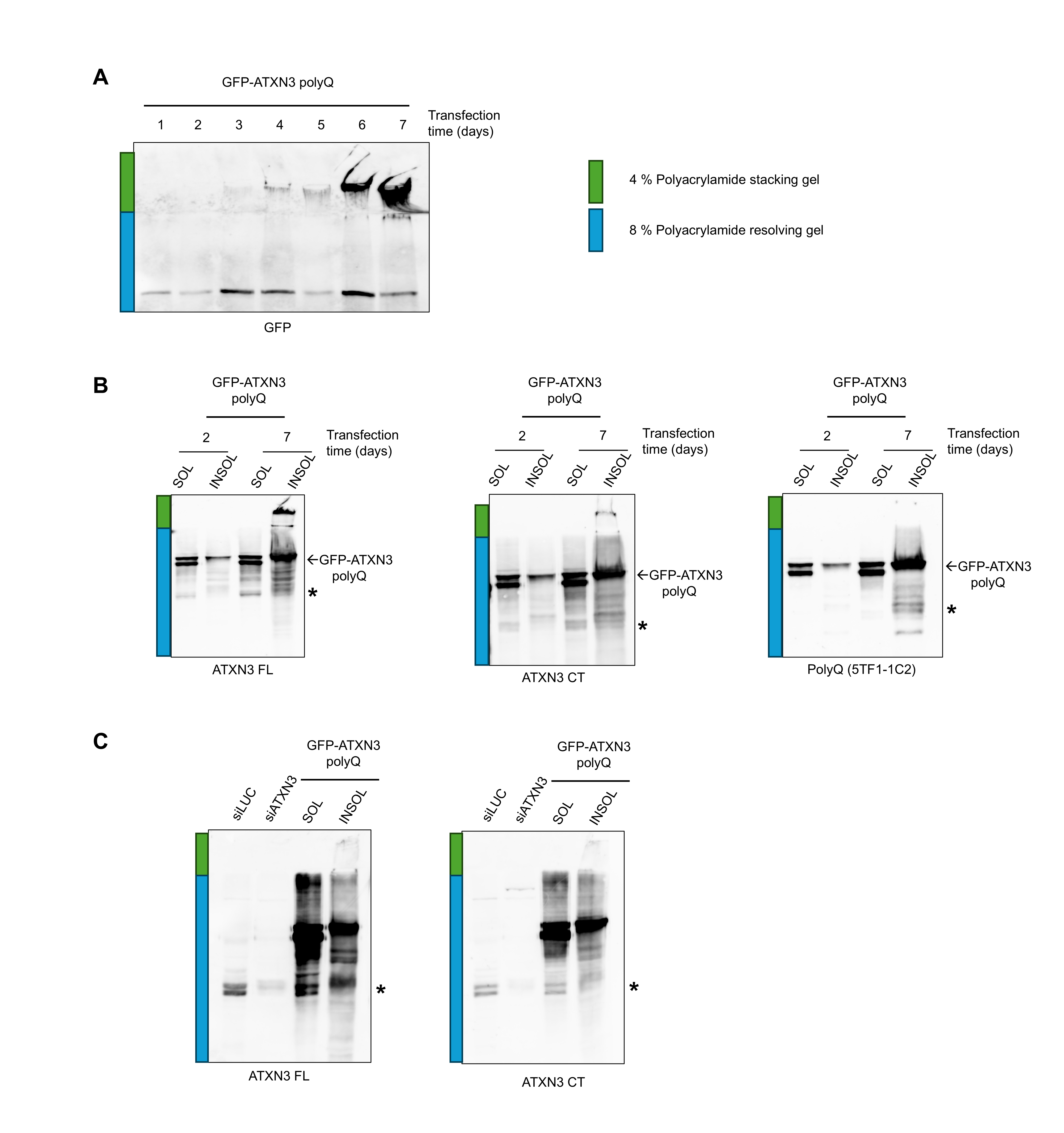

Supplement: S2 Fig — (A) HEK-239T expressing ATXN3 polyQ were lysed and fractionated at the indicated time points, followed by analysis by SDS-PAGE and immunoblot for GFP. (B) HEK-239T were transfected with GFP-ATXN3 polyQ expressing plasmid and cells were collected at the indicated times. After lysis and fractionation, samples were analysed by western blot using the indicated antibodies. (C) HEK-239T were transfected with siRNAs against luciferase (siLUC) or ATXN3 (siATXN3) for 48 hours, after which whole cell extracts were prepared. The extracts were analysed together with fractionated samples from HEK-239T transfected with GFP-ATXN3 polyQ expressing plasmid for 7 days by SDS-PAGE and western blot using the indicated antibodies. The asterisk marks the mobility of endogenous ATXN3. (TIF) [file pone.0315868.s002.tif]

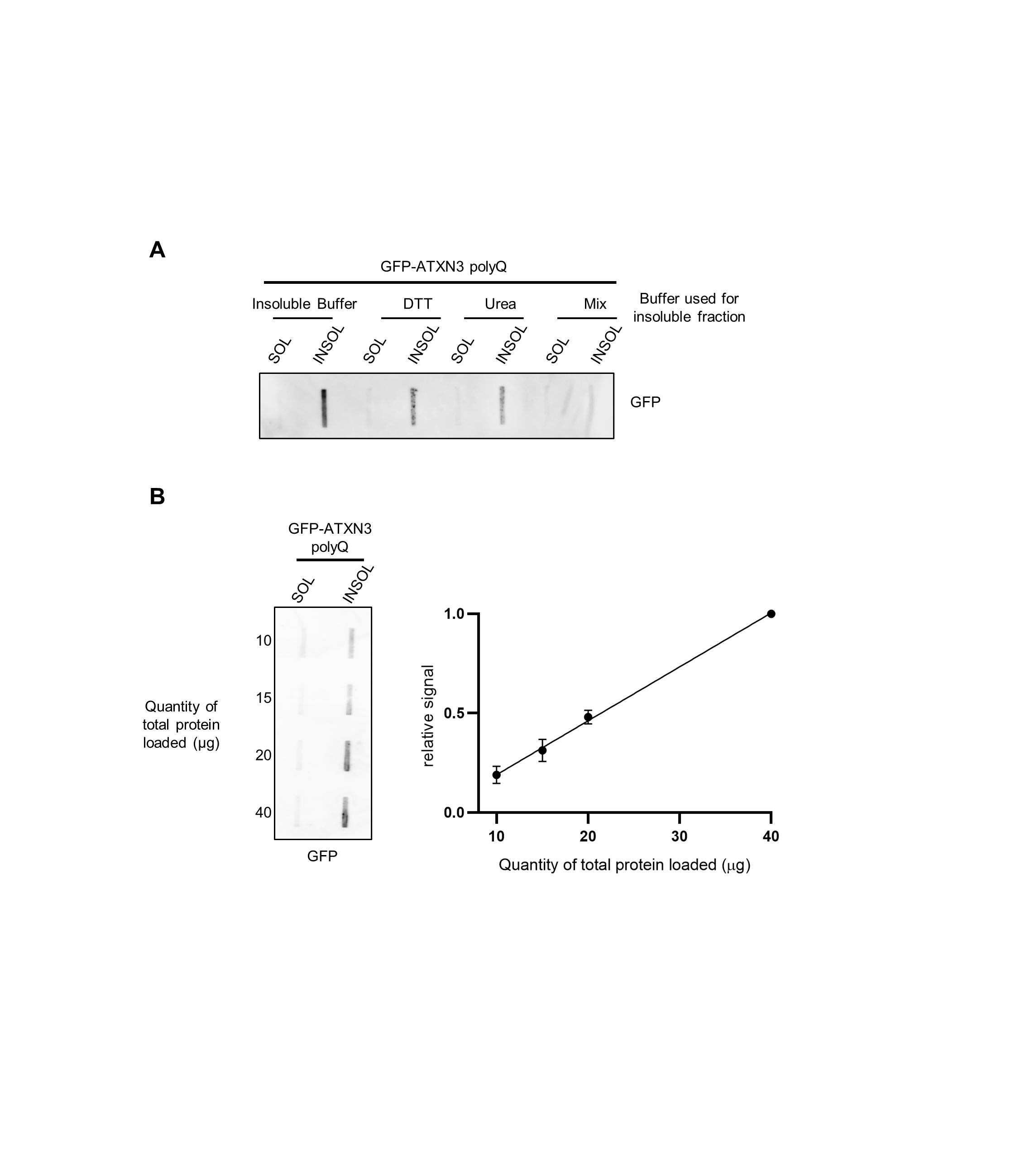

Supplement: S3 Fig — (A) HEK-239T were transfected with the GFP-ATXN3 polyQ-expressing plasmid and collected 7 days post-transfection, followed by lysis and fractionation as described in the protocol section, except for the last step. After the last wash and centrifugation, the insoluble pellet was resuspended with various buffers, including the insoluble buffer containing 4% SDS, a DTT buffer (50mM Tris 7.4, 150 mM NaCl, 100 mM DTT), an urea buffer (30 mM Tris pH 8.5, 7 M urea, 2 M thiourea) and a mix buffer (30 mM Tris pH 8.5, 7 M urea, 2 M thiourea, 100 mM DTT, 4% SDS). Soluble and insoluble fractions were subsequently analysed by FTA and immunoblotting for GFP. (B) HEK-239T expressing GFP-ATXN3 polyQ for 7 days were lysed, fractionated and analysed by FTA and immunoblot using a GFP antibody. The GFP signal in the insoluble fractions was quantified with ImageJ and plotted against the amount of protein loaded. (TIF) [file pone.0315868.s003.tif]

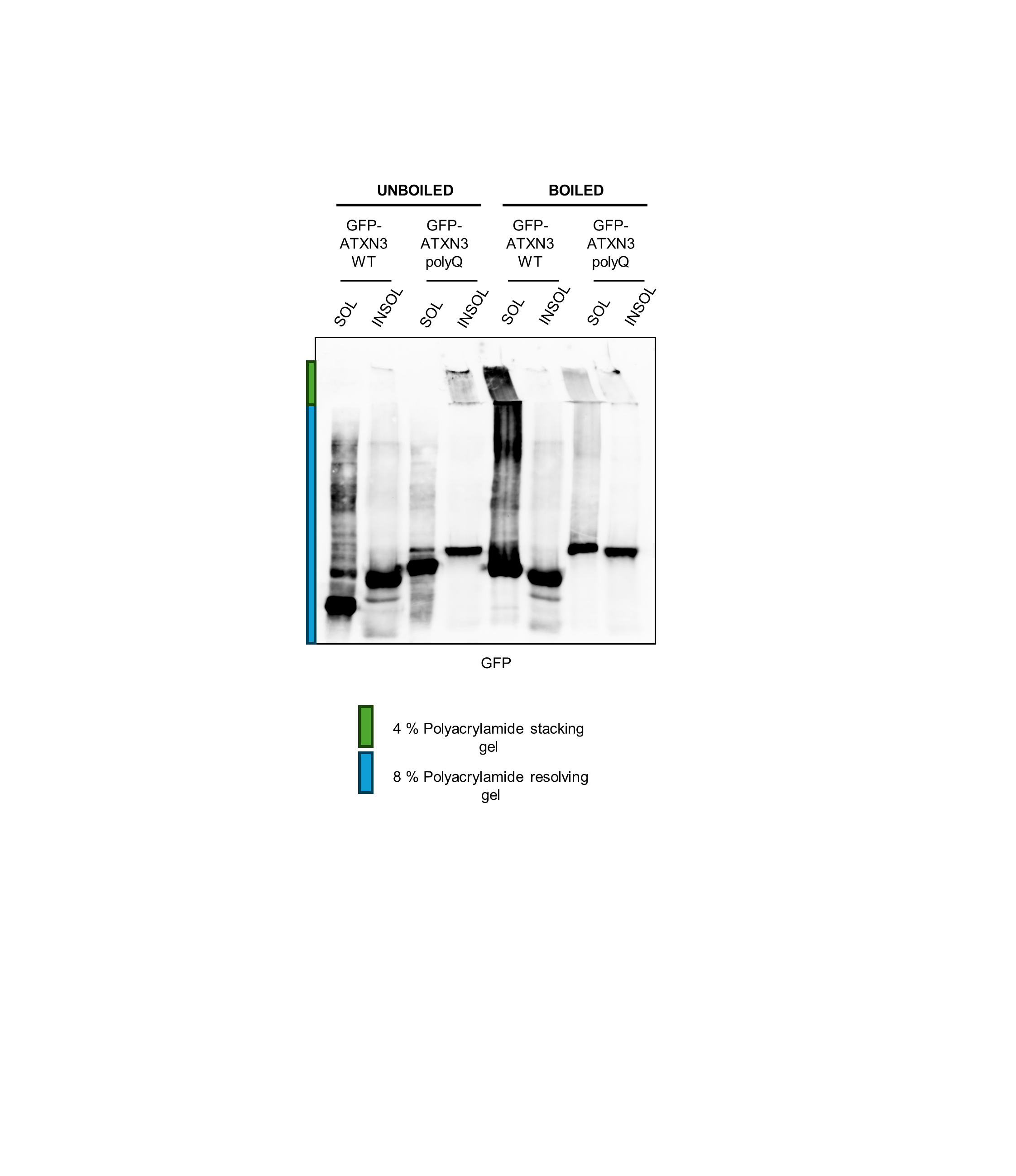

Supplement: S4 Fig — HEK-239T were transfected with the GFP-ATXN3 polyQ and WT expressing plasmids and collected at day 7 post-transfection followed by lysis, fractionation and analysis by SDS-PAGE as described in the protocol section, except that in this case samples were left untreated or boiled before loading onto the acrylamide gel, as indicated. (TIF) [file pone.0315868.s004.tif]
