# Supplementary material for: Biochemical analysis to study wild-type and polyglutamine-expanded ATXN3 species
Source: PLoS One. 2024 Dec 23;19(12):e0315868. doi: 10.1371/journal.pone.0315868 (PMC11666052; doi:10.1371/journal.pone.0315868)
Supplement: S1 File — (PDF) [file pone.0315868.s005.pdf]

Dec 06, 2024

Biochemical analysis to study wild-type and polyglutamine expanded ATXN3 species

DOI
dx.doi.org/10.17504/protocols.io.dm6gpz9q8lzp/v1

Grégoire Quinet[1,2], María Cristina Paz-Cabrera[1], Raimundo Freire[1,2,3]

[1]Fundación Canaria del Instituto de Investigación Sanitaria de Canarias (FIISC), Unidad de Investigación, Hospital Universitario de Canarias, Santa Cruz de Tenerife, Spain;
[2]Instituto de Tecnologías Biomédicas, Universidad de La Laguna, La Laguna, Spain;
[3]Universidad Fernando Pessoa Canarias, Las Palmas de Gran Canaria, Spain

Grégoire Quinet
RF lab

OPEN ACCESS

DOI: dx.doi.org/10.17504/protocols.io.dm6gpz9q8lzp/v1

Protocol Citation: Grégoire Quinet, María Cristina Paz-Cabrera, Raimundo Freire 2024. Biochemical analysis to study wild-type and polyglutamine expanded ATXN3 species. **protocols.io** https://dx.doi.org/10.17504/protocols.io.dm6gpz9q8lzp/v1

License: This is an open access protocol distributed under the terms of the **Creative Commons Attribution License**, which permits unrestricted use, distribution, and reproduction in any medium, provided the original author and source are credited

Protocol status: Working
We use this protocol and it's working

Created: June 13, 2024

Last Modified: December 06, 2024

Protocol Integer ID: 101737

Keywords: ATXN3 polyglutamine aggregation, fractionation, SDS-PAGE, Filter Trap Assay, Agarose gel electrophoresis

Funders Acknowledgement:
MCIN/AEI/10.13039/501100011033 and ERDF A way of making Europe (European Union)
Grant ID: PID2022-139691OB-I00
Fundación DISA
Grant ID: OA23/071

Abstract

Spinocerebellar ataxia type 3 (SCA3) is a cureless neurodegenerative disease recognized as the most prevalent form of dominantly inherited ataxia worldwide. The main hallmark of SCA3 is the expansion of a polyglutamine tract located in the C-terminal of Ataxin-3 (or ATXN3) protein, that triggers the mis-localization and toxic aggregation of ATXN3 in neuronal cells. The propensity of wild type and polyglutamine-expanded ATXN3 proteins to aggregate has been extensively studied over the last decades. *In vitro* studies with mass spectrometry techniques revealed a time-dependent aggregation of polyglutamine-expanded ATXN3 that occurs in several steps, leading to fibrils formation, a high status of aggregation. For *in vivo* experiments though, the techniques commonly used to demonstrate aggregation of polyglutamine proteins, such as filter trap assays, SDS-PAGE and SDS-AGE, are unable to unequivocally show all the stages of aggregation of wild type and polyglutamine-expanded ATXN3 proteins. Here we describe a systematic and detailed analysis of different known techniques to detect the various forms of both wild type and pathologic ATXN3 aggregates.

Materials

- **Plasmids and siRNAs**

GFP-ATXN3 WT: cloned in the pEGFP-C1 vector (Clontech, TaKaRa), from Hernández-Carralero et al. 2023 Nucleic Acids Res. **10.1093/nar/gkad212**1
GFP-ATXN3 polyQ: cloned in the pEGFP-C1 vector (Clontech, TaKaRa), from Hernández-Carralero et al. 2023 Nucleic Acids Res. **10.1093/nar/gkad212**
siLUC: GCACUAUUCUUGGCUCAAUdTdT ; **siATXN3:** CGUACGCGGAAUACUUCGAdTdT

- **Antibodies**

Anti-GFP: from Refolio et al. 2011 J Cell Sci. **10.1242/jcs.081711**
Anti-ATXN3 Full Lenght (FL): from Hernández-Carralero et al. 2023 Nucleic Acids Res. **10.1093/nar/gkad212**
Anti-polyglutamine/polyQ: clone 5TF1-1C2 (Sigma)
Anti-Ku86: clone B-1 (Santa Cruz, sc-5280)
Anti-ATXN3 C-Terminal (CT): Antibodies against the C-terminal part of ATXN3 were generated by injecting a rabbit with a His-tagged antigen containing the 140-361 aminoacids from ATXN3. The antigen was obtained cloning the corresponding cDNAs in pET-30 (Novagen) that was used by expression in E. Coli and purification was carried out using a Ni-NTA resin (Qiagen) following manufacturers recommendations.

- **Buffers**

All reagents were bought from Sigma except specified otherwise.
PBS (Phosphate Buffered Saline): 137 mM NaCl (Sigma-Aldrich, S3014), 2.7 mM (KCl Sigma-Aldrich, P9541), 8 mM Na_2HPO_4 (Sigma-Aldrich, S9763), 2 mM KH_2PO_4 (Sigma-Aldrich, P0662) pH 7.4.
TBS (Tris-Buffered Saline): 20 mM Tris (Sigma-Aldrich, T1503) -HCl (Sigma-Aldrich, 258148) pH8, 150 mM NaCl.
TBS-T: TBS containing 1% Tween-20 (Sigma-Aldrich, P5927).
TBS-T + 5% milk: TBS-T containing 5% skimmed milk powder.
TBS-T + 2.5% milk: TBS-T containing 2.5% skimmed milk powder.
Soluble Buffer: 10 mM Tris pH 7.4, 1% Triton-X 100 (Sigma-Aldrich, T8787), 175 mM NaCl, 10% glycerol (Sigma-Aldrich, 49781)
Soluble Buffer supplemented with inhibitors: 10 mM Tris pH 7.4, 1% Triton-X 100, 175 mM NaCl, 10% glycerol, 1:1000 of Protease Inhibitor Cocktail Set III (Calbiochem, 535140), 1 mM phenylmethylsulphonyl fluoride (PMSF, ITW Reagents, A0999) in 2-propanol (VWR Chemicals, 20839.366), 2.5 mM NaF (Sigma-Aldrich, 201154), 2 mM *N-Ethylmaleimide* (NEM, Sigma-Aldrich, 04259), 100U/ml Universal nuclease for Cell Lysis (Pierce, 88702)
Insoluble Buffer: Soluble Buffer + 4% Sodium dodecyl sulfate (SDS, Sigma-Aldrich, L4509).
Laemmli buffer: 62.5 mM Tris-HCl pH 6.8, 10% glycerol, 2% SDS, 5% *2-Mercaptoethanol (*Sigma-Aldrich*, M3148)*, 0.005% Bromophenol Blue
SDS-PAGE electrophoresis Buffer: 192 mM glycine (Sigma-Aldrich, G8898), 25 mM Tris pH 8, 0.1% SDS
SDS-PAGE transfer buffer: 192 mM glycine, 25 mM Tris pH 8, 20% ethanol
AGE Loading buffer: 40 mM Tris pH 7.8, 1% SDS, 30% glycerol, 1mM EDTA, 0.005% Bromophenol Blue (Sigma-Aldrich, 114391)
Agarose gel buffer: 20% Glycerol, 1 mM Ethylenediaminetetraacetic acid pH 8 (EDTA, Sigma-Aldrich, E9884), 50 mM Tris pH 8, 0.1% SDS, 0.38 M glycine.
SDS-AGE electrophoresis buffer: 40 mM Tris pH 8, 0.1% SDS, 1 mM EDTA
SDS-AGE transfer buffer: 0.05 M Glycine, 7 mM Tris pH 8.3, 0.025% SDS
FTA buffer: PBS + 1% SDS

HBS: 250 mM NaCl, 1.5 mM Na2HPO4, 12 mM glucose (Sigma-Aldrich, G8270), 10 mM KCl, 50 mM HEPES (Sigma-Aldrich, H4034), adjust pH at 7.02.

Cell Culture

1 Plate HEK-293T cells in 10 cm dish plates to be 15-20 % confluent and wait around 4 hours for the cells to attach.

2 Mix 3 µg of ATXN3 plasmid with 50 µl of 3.3 M $CaCl_2$ (Sigma-Aldrich, 208291) and 450 µl of filtered mQ water. Mix with 500 µl **HBS**

3 Add quickly to the cells and mix well. Incubate overnight.

4 The next day, wash cells with fresh medium.

5 Let cells express the transfected ATXN3 species for 7 days, renew culture medium after 5 days.

NOTE: time of expression can be adapted. In our hands, aggregation of SDS-insoluble high molecular weight ATXN3-polyQ starts from 3-4 days and is optimal after 7 days of transfection with the GFP-ATXN3 polyQ plasmid.

Fractionation Assay

6 Wash three-time cells with **PBS**, collect, count and transfer between 5 to 10 x10^6 cells in a 1.5 ml low protein binding tube.

7 Centrifuge 5 minutes at 300 g at room temperature, discard PBS.

8 For lysis, resuspend the pellet with 500 µl of **Soluble Buffer supplemented with inhibitors** and incubate during one hour on ice.

9 Centrifuge for 20 min at 15.000 g at 4°C.

10 Recover supernatant as the soluble fraction in a 1.5 ml tube. Sonicate the soluble fraction (6x 30 s sonication; 80% power; 30 s pulse/30 s pause; 4°C).

11 Wash pellet with 1ml **Soluble Buffer.**

12 Vortex until pellet detaches from the bottom, centrifuge for 5 min at 15.000 rpm 4ºC, remove carefully all the supernatant.

13 Repeat the two previous steps once more.

14 Resuspend pellet in 250 µl of **Insoluble Buffer**.

15 Sonicate (18x 30 s sonication; 80 % power; 30 s pulse/ 30 s pause; 4ºC).

16 Boil insoluble fraction for 5 min, pulse centrifuge and vortex well.

17 Quantify the protein amount in soluble and insoluble Fractions, for example with BCA (Pierce bca protein assay kit, 23225).

18 Store samples at -20ºC.

SDS-Polyacrylamide Electrophoresis

19 Thaw the soluble fraction samples on ice and incubate the insoluble fractions at 37ºC until the SDS is solubilized. Vortex well.

20 Prepare 10-40 µg of total proteins for both fractions in new 1.5 ml tubes. Adjust the quantity of proteins in the insoluble fraction samples to load relative to their corresponding soluble fractions.

21 Add 1:3 v/v of **Laemmli buffer** to every sample.

22 Load soluble fraction with their respective insoluble fractions onto an 8 % acrylamide:bisacrylamide (29:1) (ITW Reagents, A0951) gel with a 4 % acrylamide:bisacrylamide (29:1) stacking gel.

23 Separate the proteins by electrophoresis in **SDS-PAGE electrophoresis** buffer for 1 hour at 180 V.

24 Transfer the proteins of the entire gel, including the stacking and the wells, in **SDS-PAGE transfer buffer** onto a nitrocellulose membrane (Cytiva, 10600003) for 2.5 hours at 300 mA.

25 Process by immuno-blotting as described in the corresponding section.

SDS-Agarose Gel Electrophoresis

26 Thaw the soluble fraction samples on ice and incubate the insoluble fractions at 37ºC until the SDS is solubilized. Vortex well.

27 Prepare 10-40 µg of total proteins for both fractions in new 1.5 ml tubes. Adjust the quantity of proteins in the insoluble fraction samples to load relative to their corresponding soluble fractions.

28 Add 1:3 v/v µl of **AGE loading buffer** in every sample.

29 Mix 0.4 g of agarose (VWR Chemicals, 438792U) with 40 ml of **agarose gel buffer**, boil and mix until complete resuspension of the agarose.

30 Wait until the agarose gel buffer cools until approximately 60ºC, and load into a 1.5mm spaced plate set in an SDS-PAGE cassette, add the comb and wait for complete cooling and polymerization.

NOTE: A 1cm 8% acrylamide plug can be prepare before the agarose in the bottom of the gel. It will help the gel to stay in the plate set and ease comb extraction and gel manipulation.

31 Remove the combs. A previous incubation of the gel for 10 min at 4ºC helps the removal of the comb.

32 Load the soluble and insoluble fractions.

33 Separate the proteins by electrophoresis on ice in **SDS-AGE electrophoresis buffer** for approximatively 1 hour at 80V.

34 Transfer the proteins onto a nitrocellulose membrane in **SDS-PAGE transfer buffer** overnight at 100mA at 4ºC.

35 Process by immuno-blotting as described in the corresponding section.

Filter Trap Assay

36 Thaw the soluble fraction samples on ice and incubate the insoluble fractions at 37°C until the SDS is solubilized. Vortex well.

37 Prepare 10-60 µg of total proteins for both fractions in new 1.5 ml tubes. Adjust the quantity of proteins in the insoluble fraction samples to load relative to their corresponding soluble fractions.

38 Complete until 120 ul with **FTA buffer**. Depending on the protein of interest signal, quantity of proteins loaded can be adjusted.

NOTE: in this section volumes of samples and washes are adapted for the BioSlot Blot SF machine from Bio-Rad (1706542), it can be adjusted to fit to other apparatus.

39 Pre-soak filter paper and acetate membrane (Cytiva, 10404180) in 50ml **FTA buffer** for 10 min.

40 Assemble the cassette with the filter/membrane, thereby avoiding bubbles, tighten the screws in a diagonal order, apply vacuum and tighten again to avoid leaking.

41 Load 100 µl of **FTA buffer** in every well, apply vacuum until no liquid remains. Beware to not let the membrane dry.

42 Load 120 µl of each sample, apply vacuum until all samples pass through the filter. Note that if some liquid remains after 5 min of vacuum, it could mean that the filter is saturated. In this case, the quantity of protein loaded must be reduced.

43 Wash twice by loading 100 ul of **FTA buffer** and applying vacuum until the liquid has passed through.

44 Disassemble the cassette, rinse the acetate membrane in milliQ water.

45 Process by immuno-blotting as described in the corresponding section.

Immuno-blotting

46 Block the nitrocellulose or acetate membrane in **TBS-T + 5% milk** for 1 h at room temperature with agitation.

47 Incubate the membrane with the adequate primary antibody diluted in **TBS-T + 2.5 % milk** for 1 hour at room temperature or overnight at 4ºC. In our case we used anti-GFP (1:500) to specifically visualize GFP-ATXN3.

48 Wash the membranes 3 times for 10 min in **TBS-T**.

49 Incubate the membranes with HRP-conjugated secondary antibody diluted in **TBS-T + 2.5 % milk** for one hour at room temperature.

50 Wash the membranes in **TBS-T** 3 times for 10 min.

51 Visualize blots using chemiluminescent substrate (Thermoscientific, product #34577) using an ImageQuant LAS 4000 or equivalent.
