# Supplementary material for: Biochemical analysis to study wild-type and polyglutamine-expanded ATXN3 species
Source: PLoS One. 2024 Dec 23;19(12):e0315868. doi: 10.1371/journal.pone.0315868 (PMC11666052; doi:10.1371/journal.pone.0315868)
Supplement: S1 Raw images — (PDF) [file pone.0315868.s006.pdf]

Fig 1A

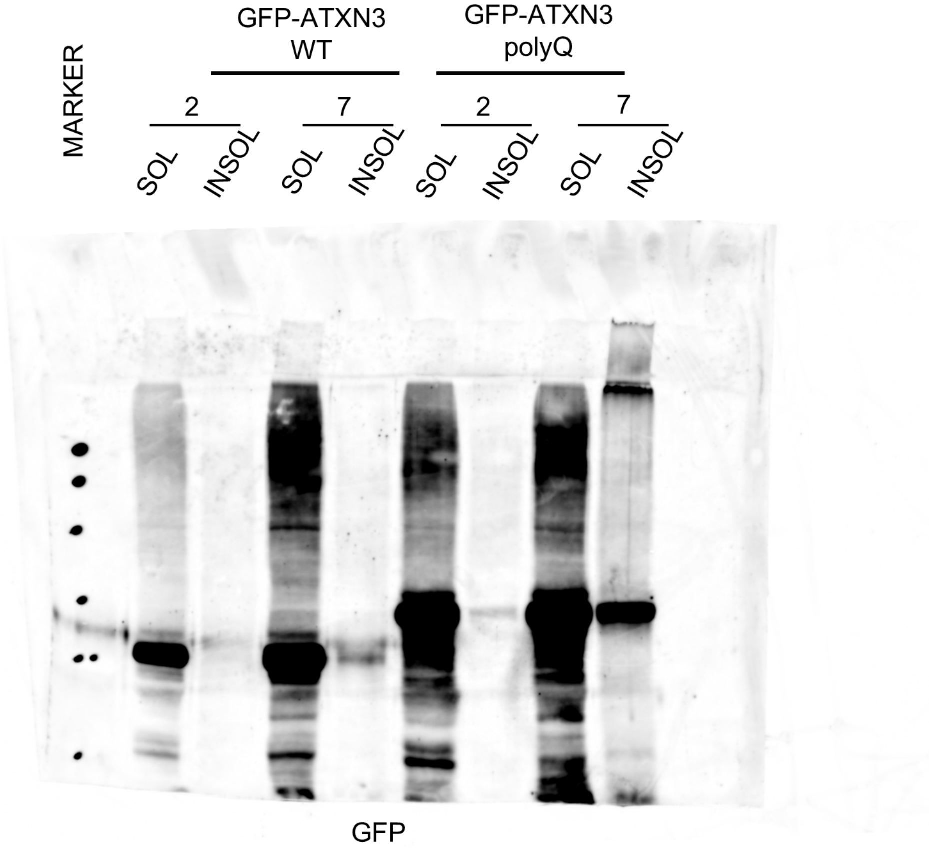

MARKER
GFP-ATXN3
WT
GFP-ATXN3
polyQ
2
7
2
7
SOL
INSOL
SOL
INSOL
SOL
INSOL
SOL
INSOL
GFP

Fig 1B

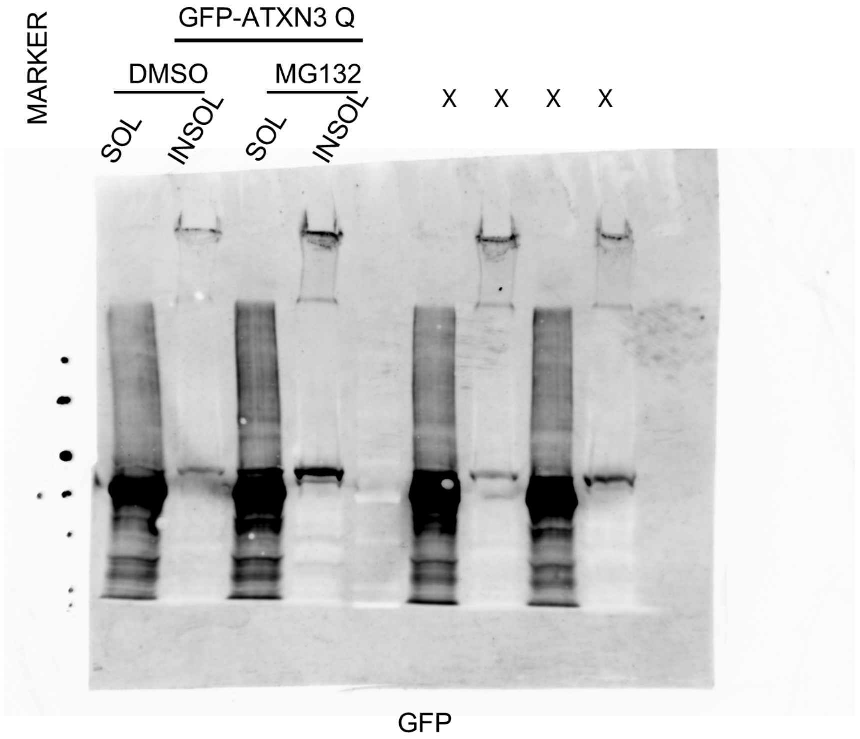

MARKER
GFP-ATXN3 Q
DMSO
MG132
SOL
INSOL
SOL
INSOL
X
X
X
X
GFP

Fig 2A

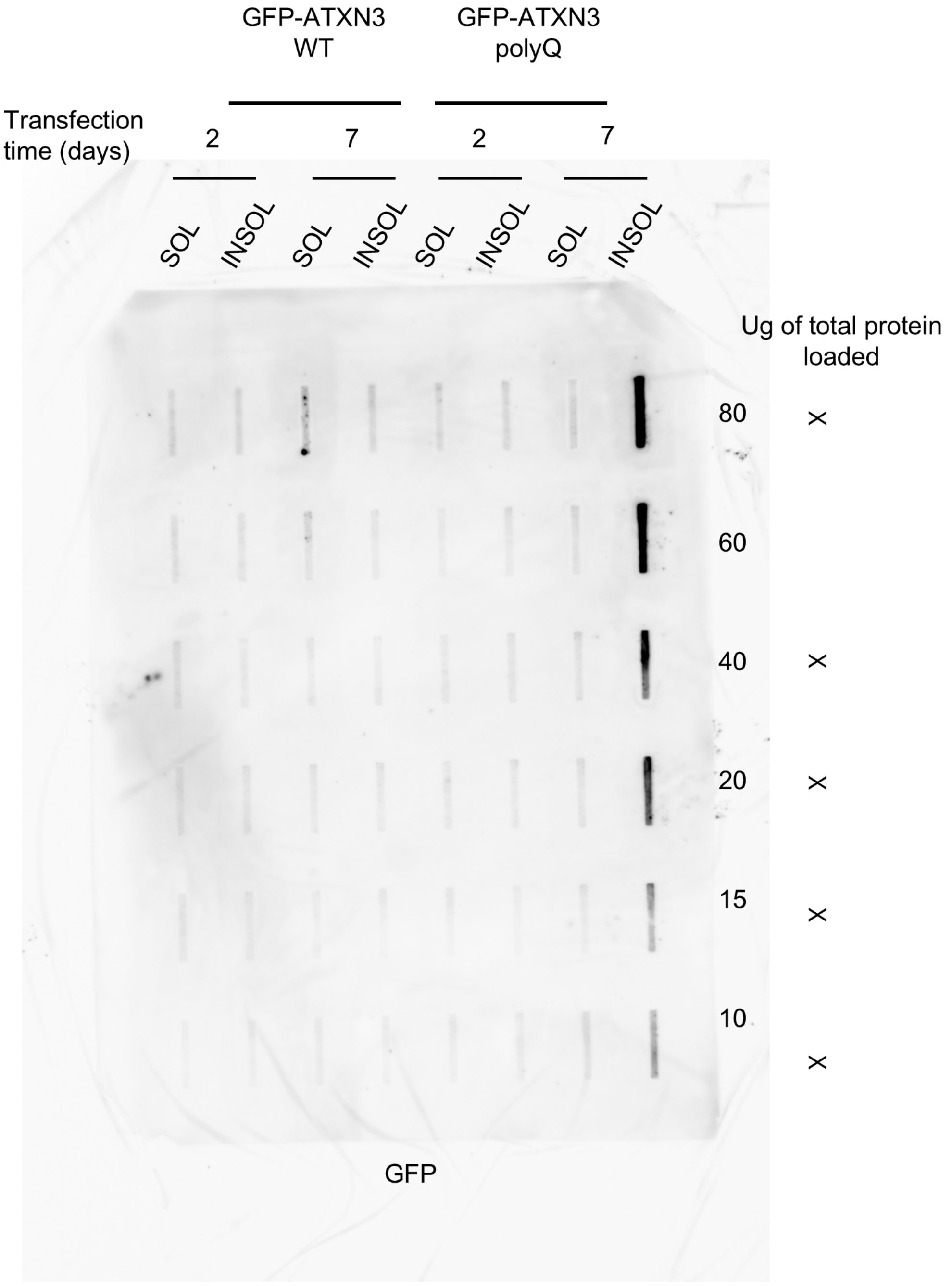
GFP-ATXN3 WT
GFP-ATXN3 polyQ
Transfection time (days)
2
7
2
7
SOL
INSOL
SOL
INSOL
SOL
INSOL
SOL
INSOL
Ug of total protein loaded
80
60
40
20
15
10
GFP

Fig 2B

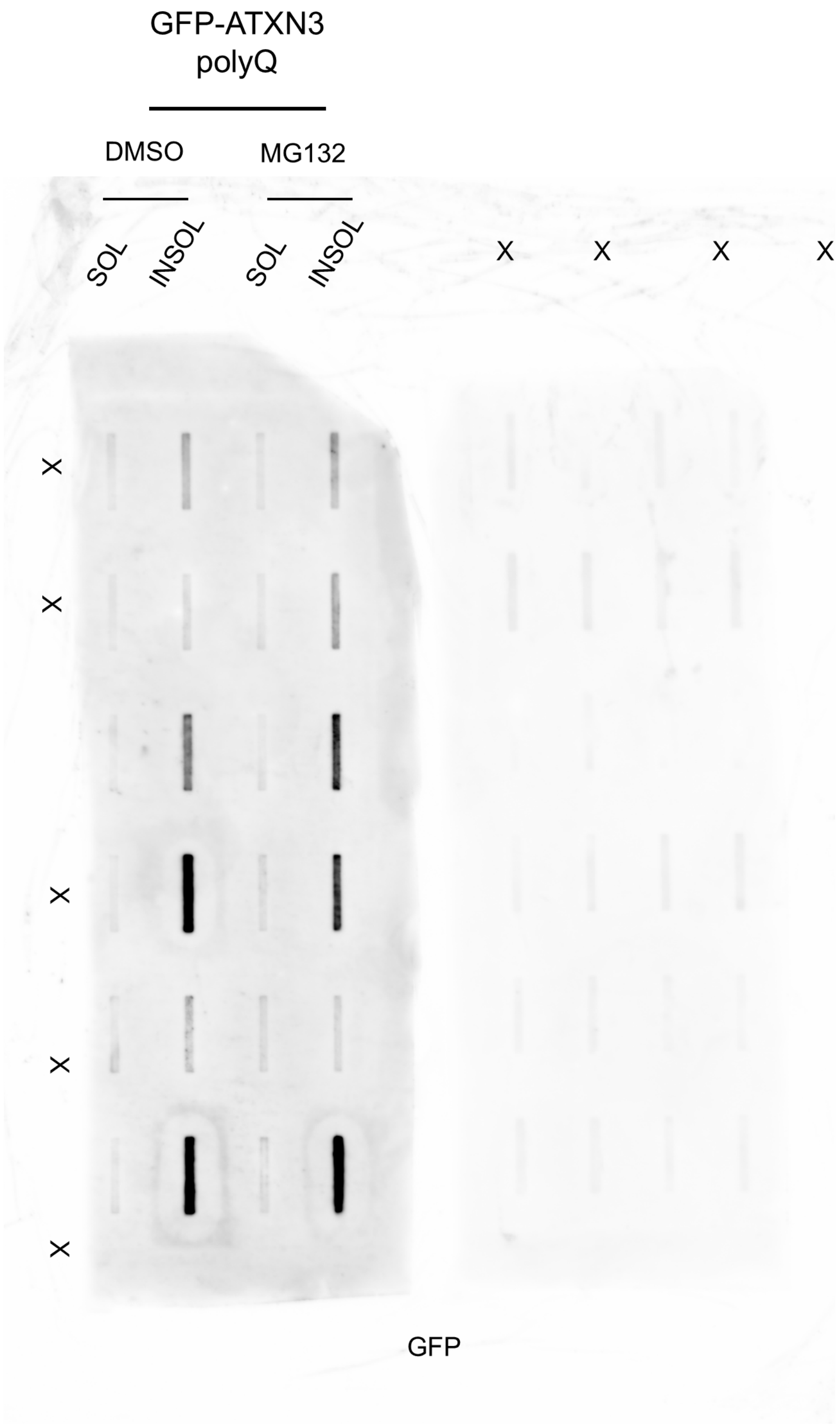
GFP-ATXN3
polyQ
DMSO
MG132
SOL
INSOL
SOL
INSOL
X
X
X
X
X
X
X
X
X
GFP

Fig 3A

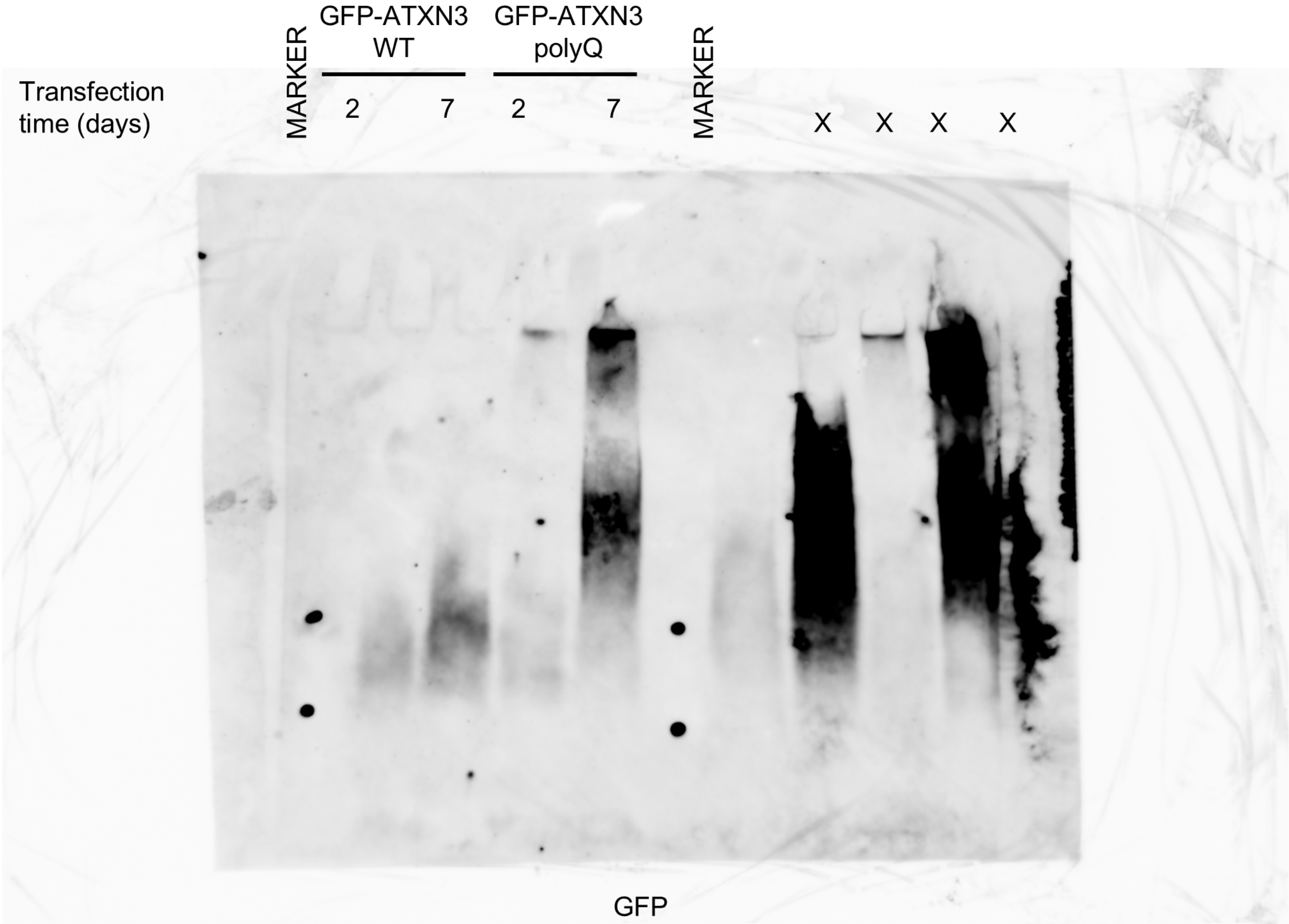
GFP-ATXN3
WT
GFP-ATXN3
polyQ
Transfection
time (days)
MARKER
2
7
2
7
MARKER
X
X
X
X
GFP

Fig 3B

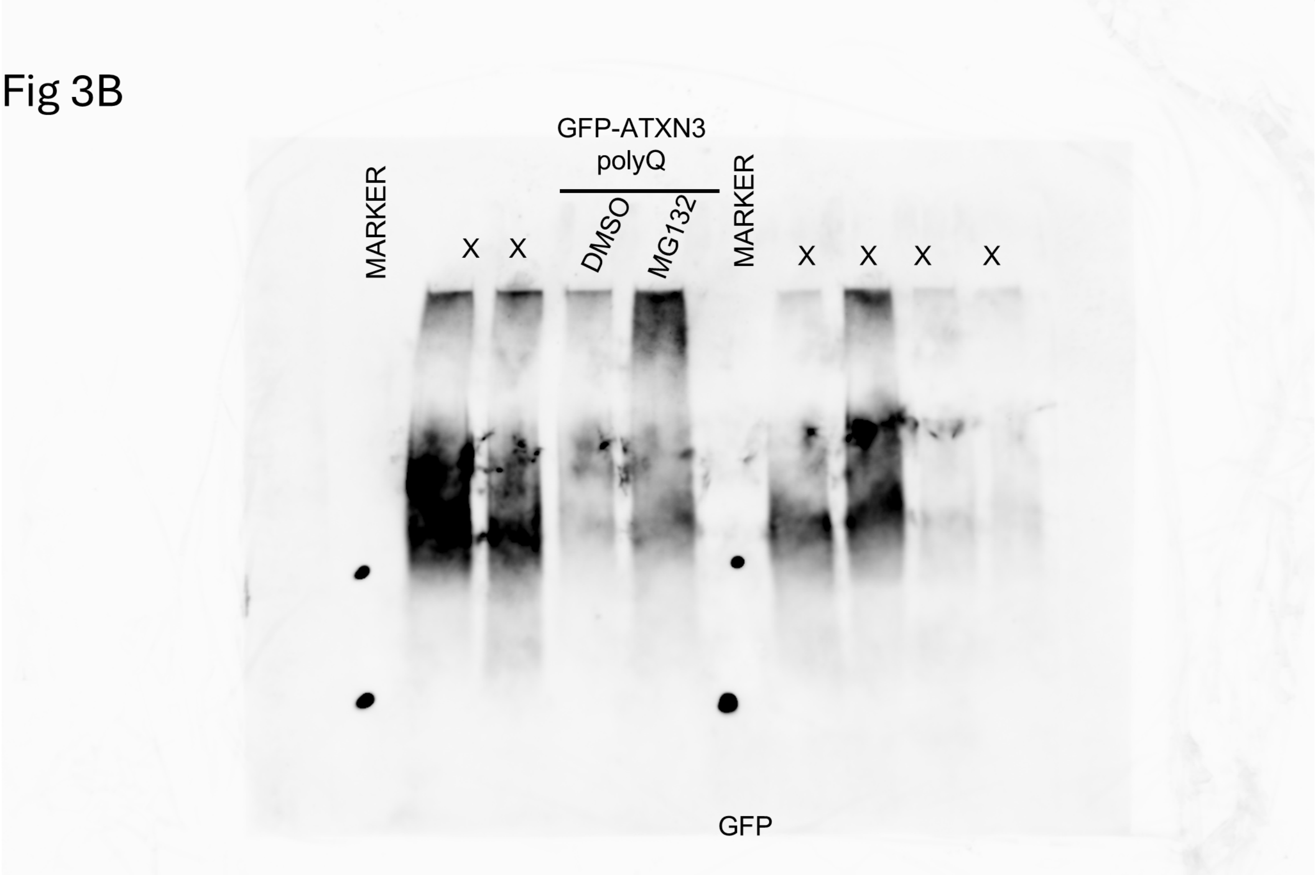
GFP-ATXN3
polyQ
MARKER
X
X
DMSO
MG132
MARKER
X
X
X
X
GFP

S1 FigA

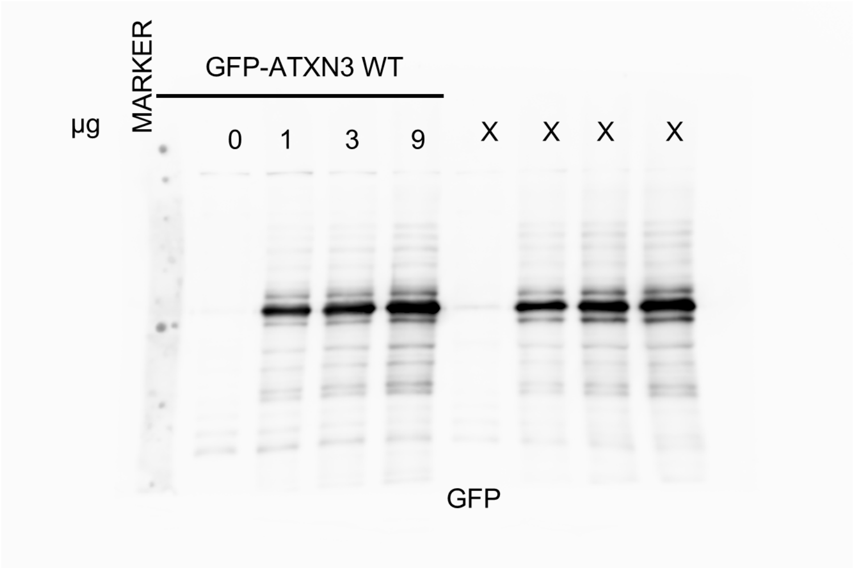
MARKER
GFP-ATXN3 WT
µg
0 1 3 9
X X X X
GFP

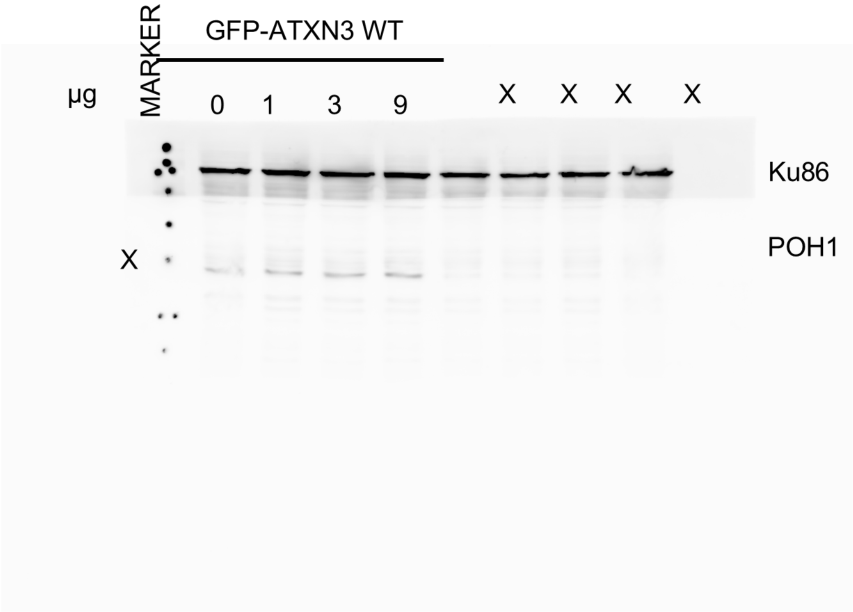
MARKER
GFP-ATXN3 WT
µg
0 1 3 9
X X X X
Ku86
POH1
X

S1 FigB

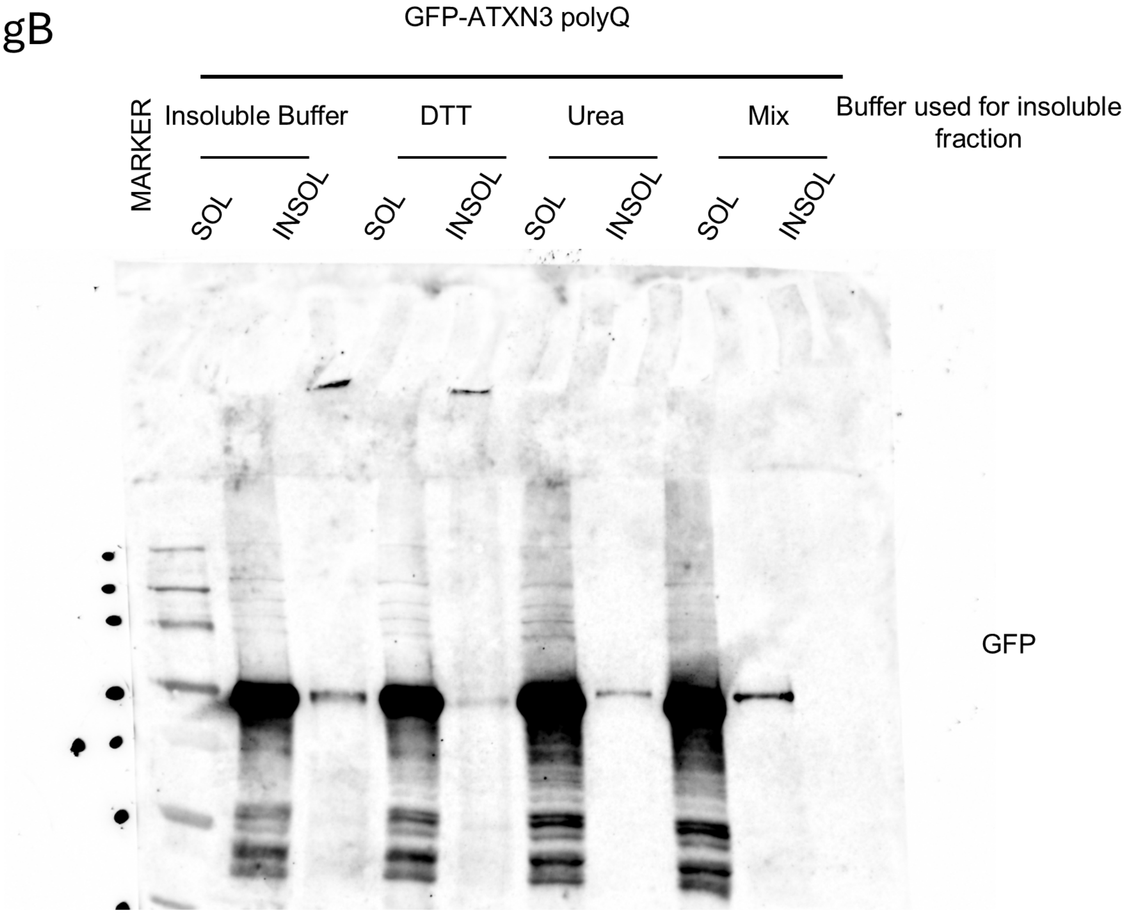
GFP-ATXN3 polyQ
MARKER
Insoluble Buffer
DTT
Urea
Mix
Buffer used for insoluble fraction
SOL
INSOL
SOL
INSOL
SOL
INSOL
SOL
INSOL
GFP

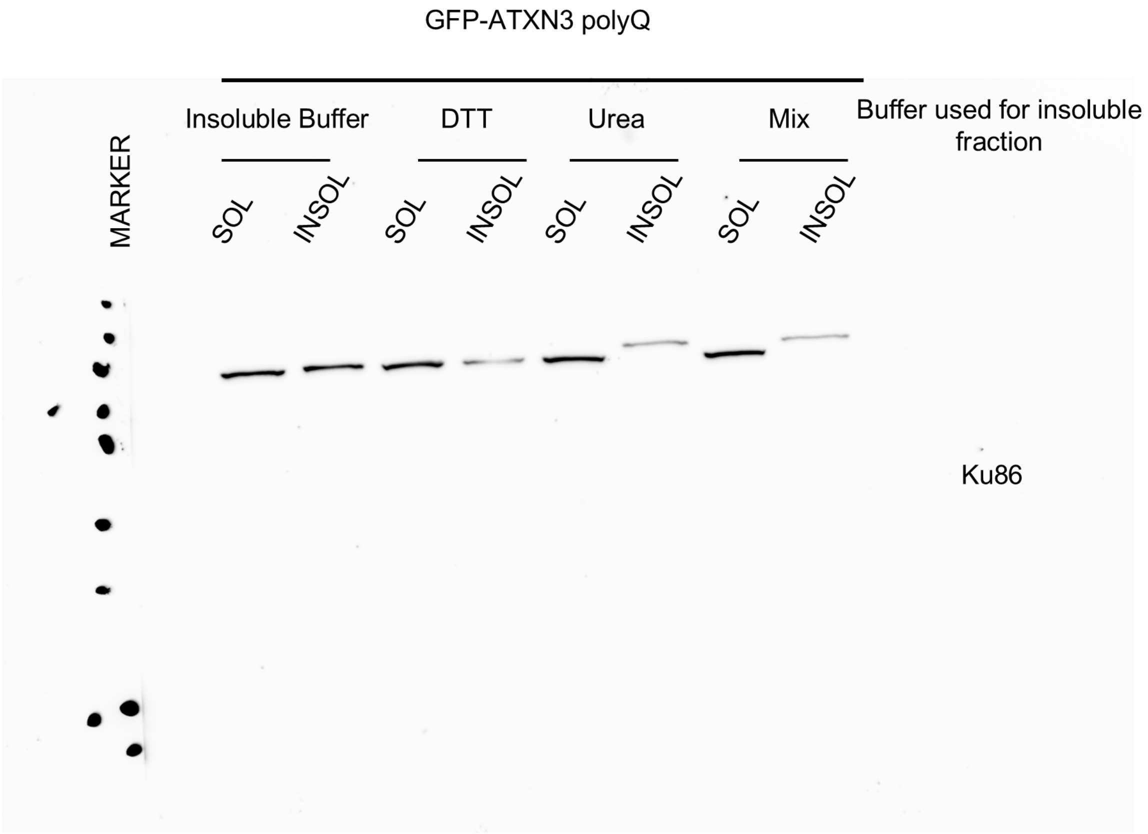
GFP-ATXN3 polyQ
MARKER
Insoluble Buffer
DTT
Urea
Mix
Buffer used for insoluble fraction
SOL
INSOL
SOL
INSOL
SOL
INSOL
SOL
INSOL
Ku86

S1 FigC left panel

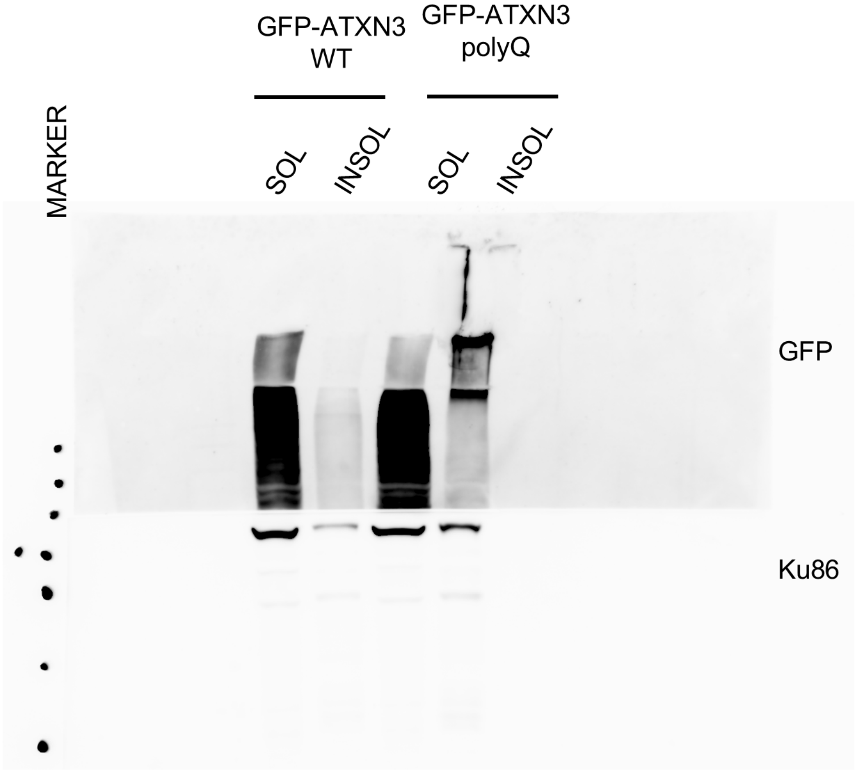

S1 FigC right panel

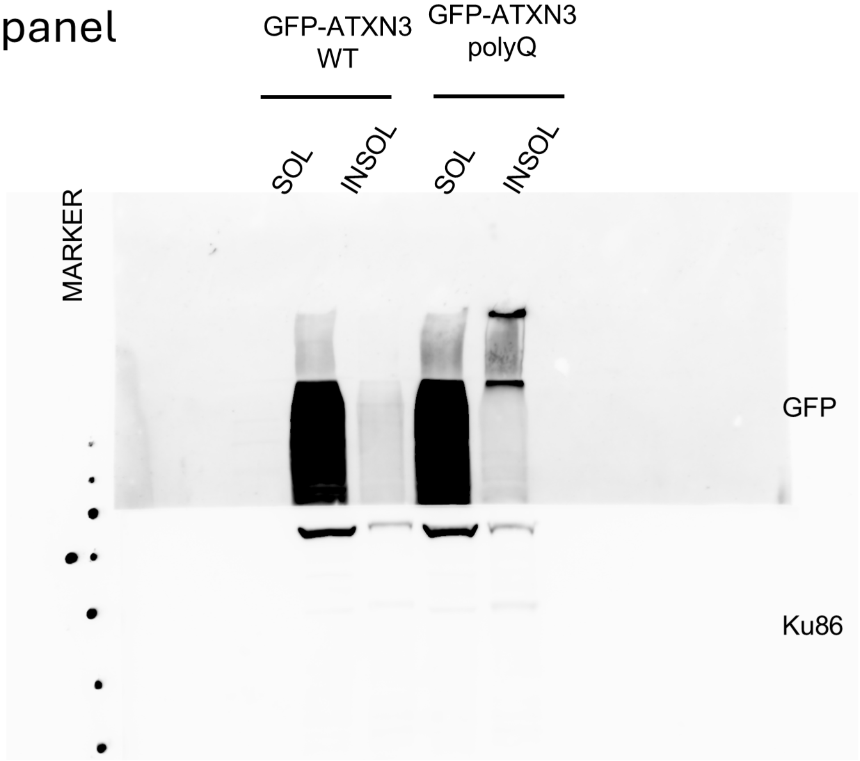

S1 FigD

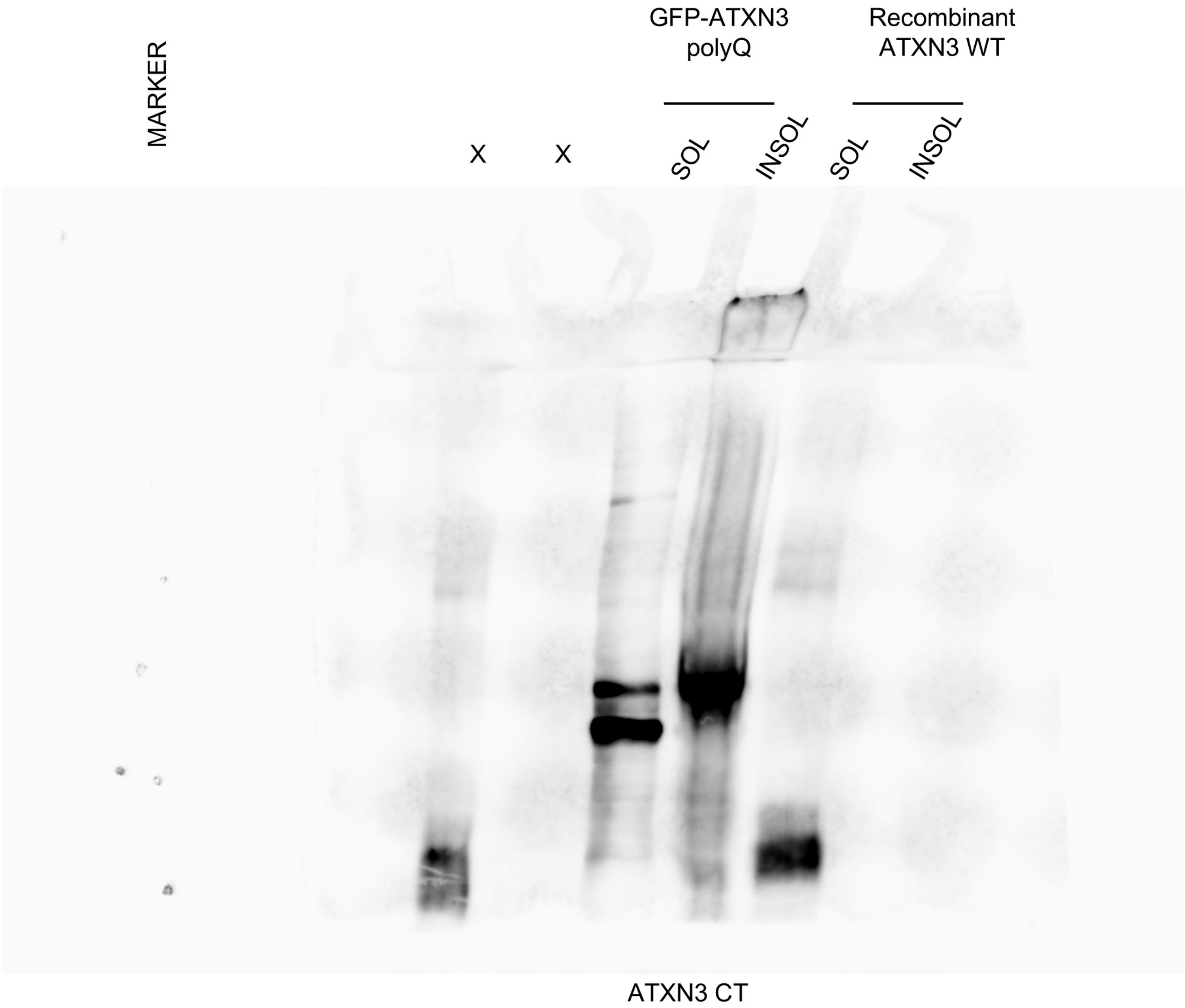
GFP-ATXN3
polyQ
Recombinant
ATXN3 WT
MARKER
X
X
SOL
INSOL
SOL
INSOL
ATXN3 CT

S1 FigE

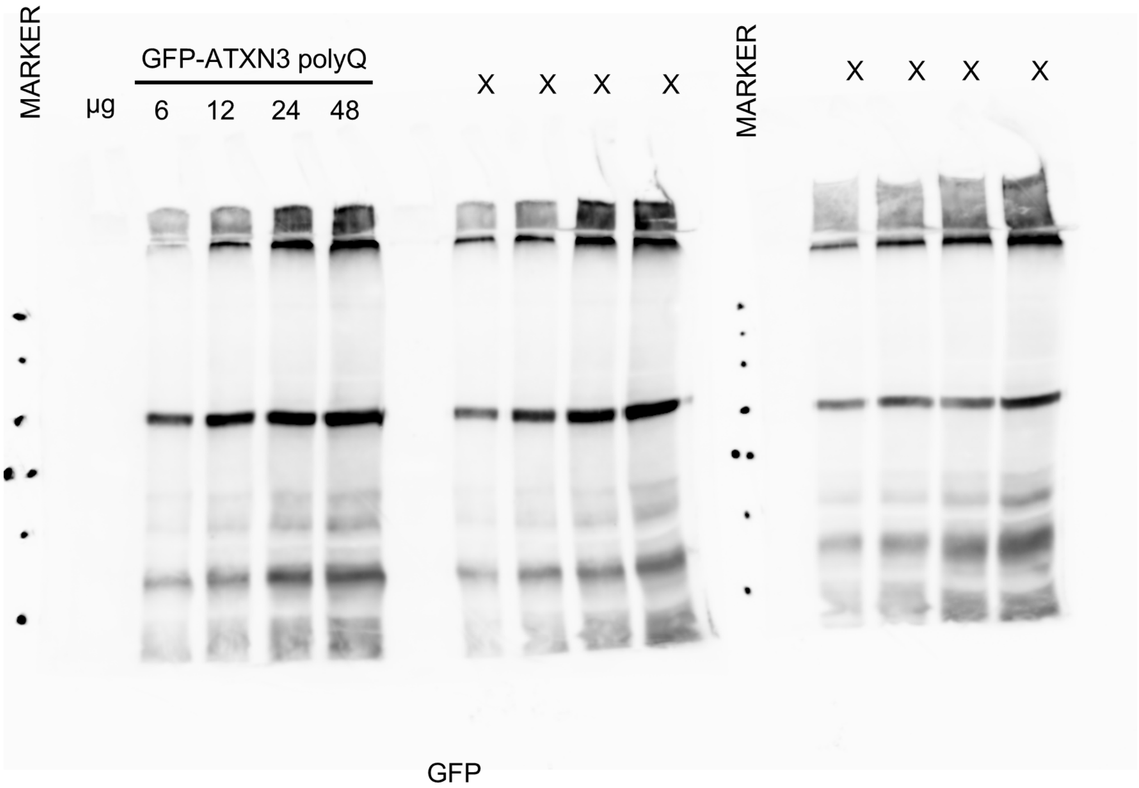

S2 FigA

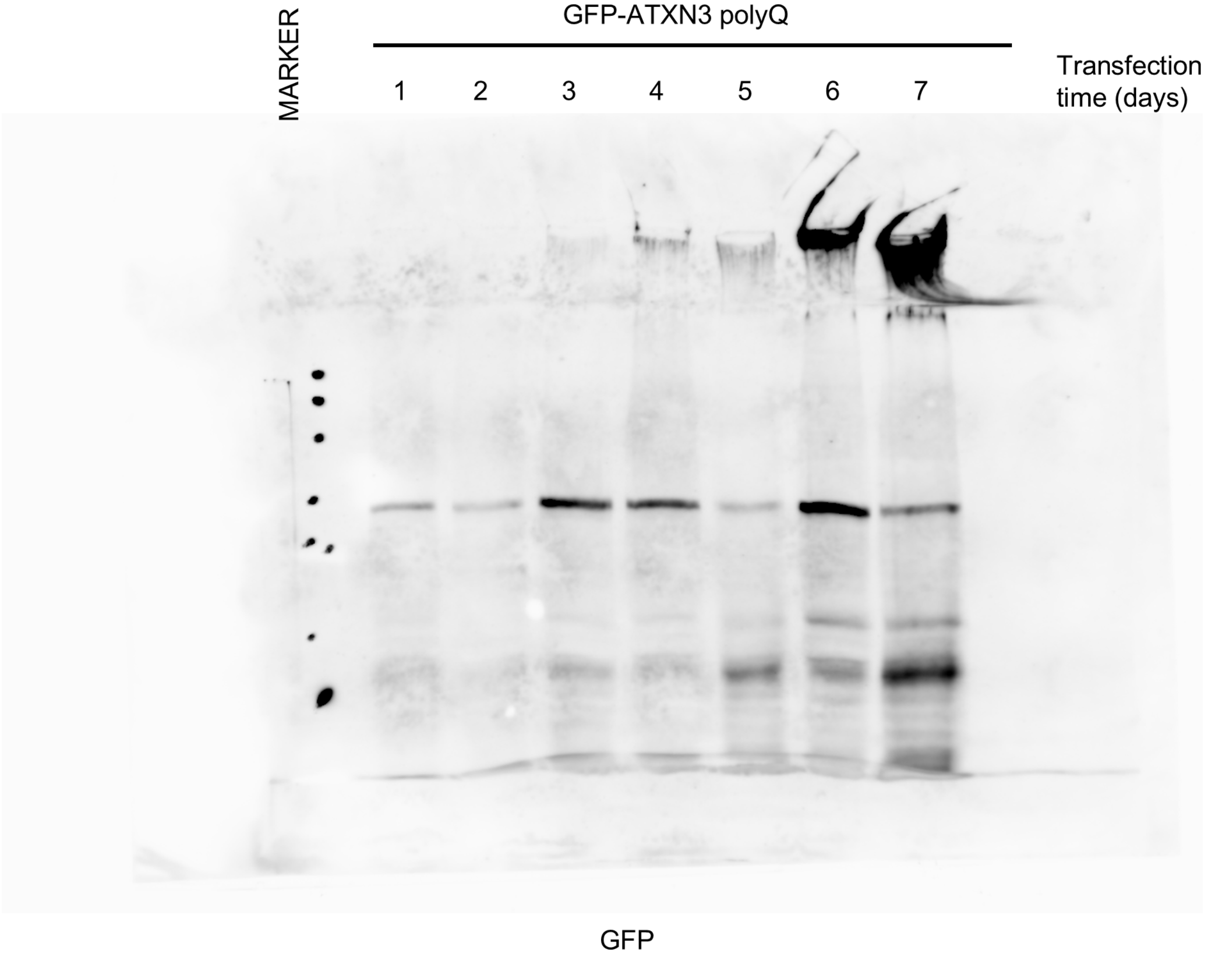

S2 FigB left panel

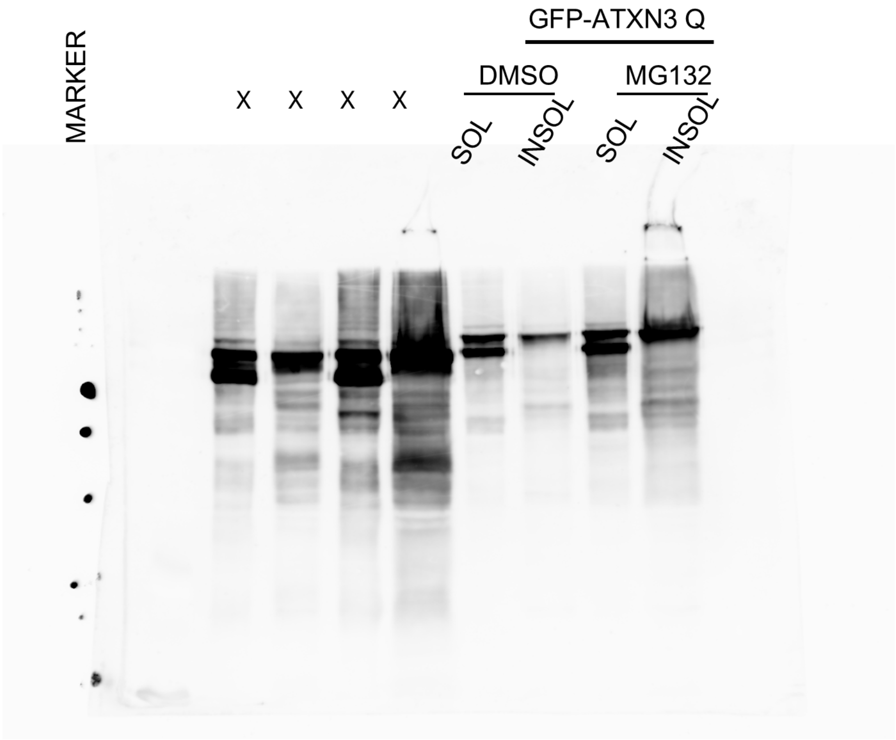

S2 FigB middle panel

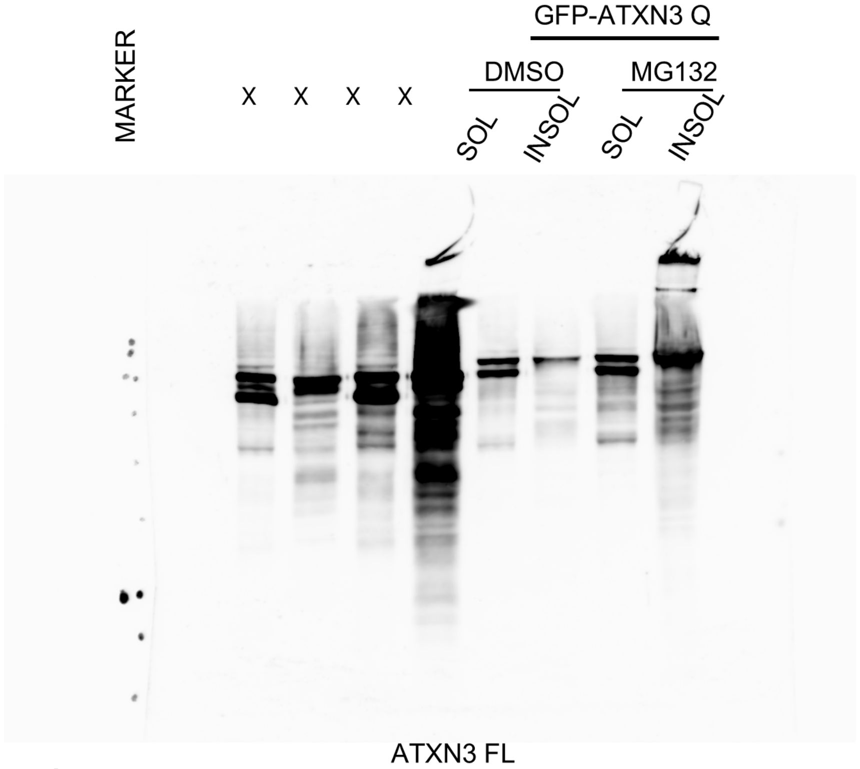

S2 FigB right panel

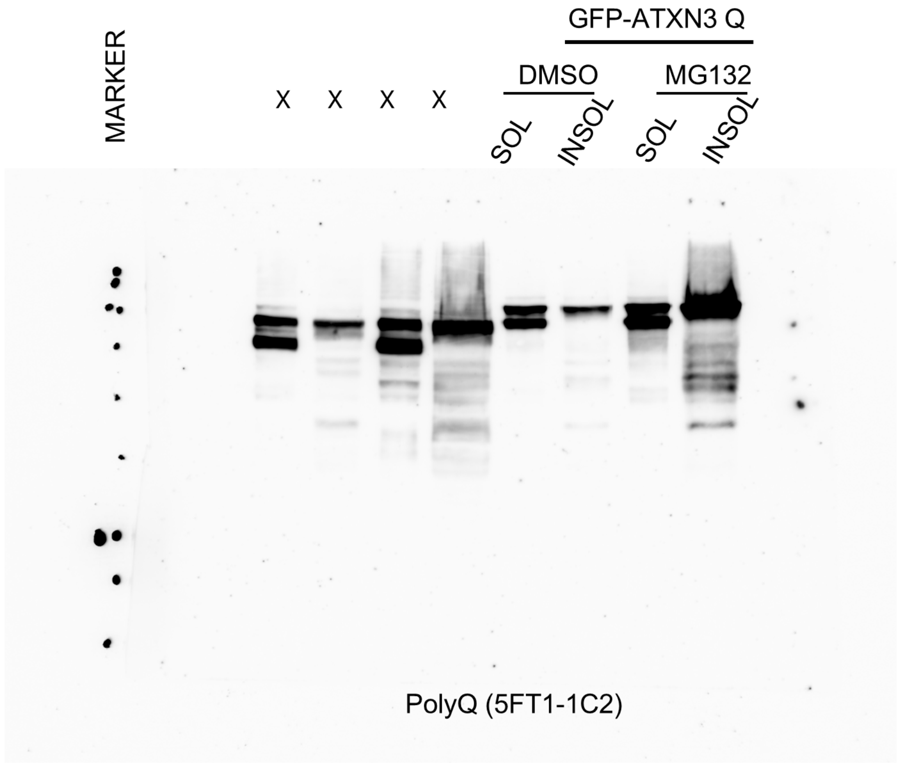

S2 Fig C left and right panels

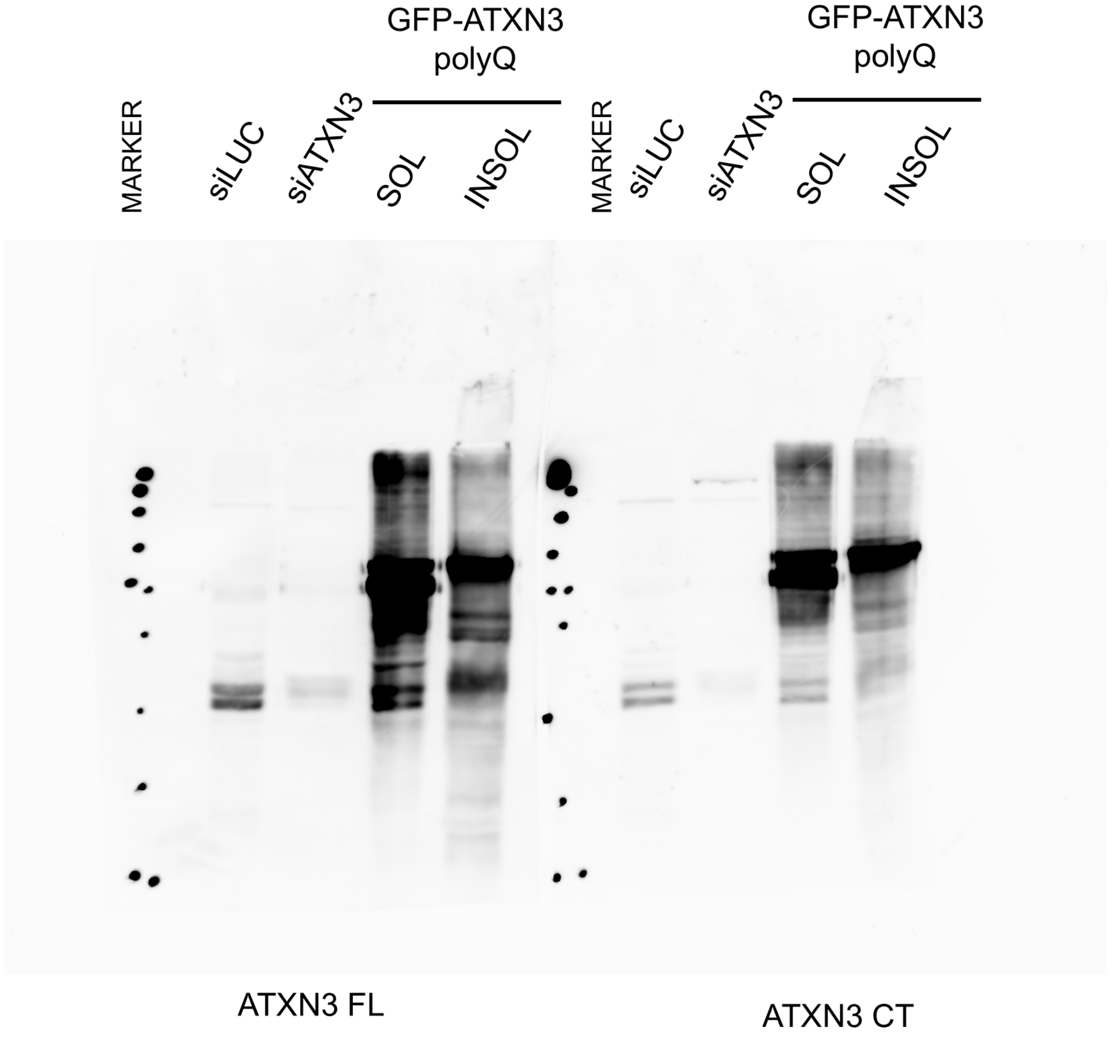

S3 FigA

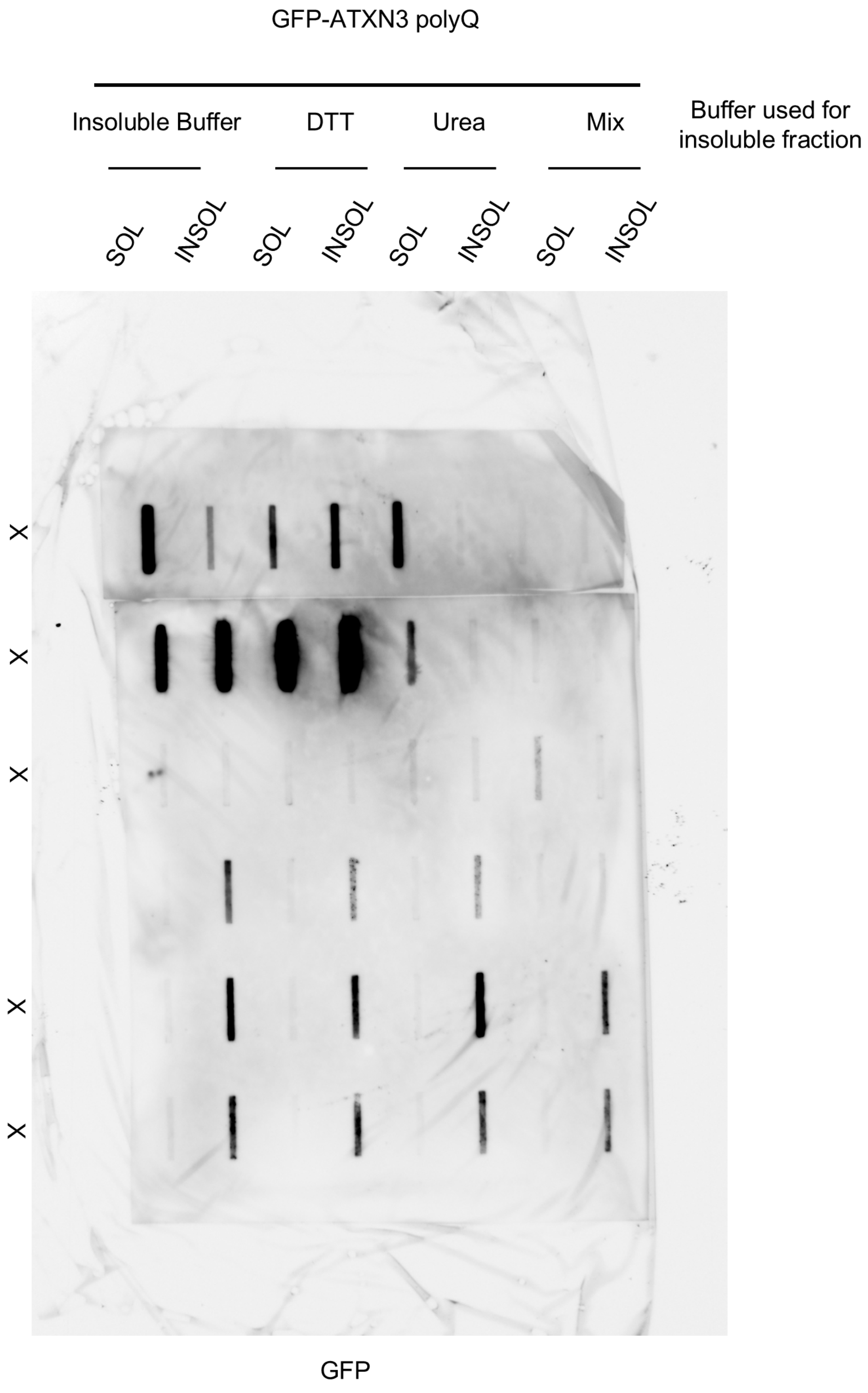
GFP-ATXN3 polyQ
Insoluble Buffer
DTT
Urea
Mix
Buffer used for insoluble fraction
SOL
INSOL
SOL
INSOL
SOL
INSOL
SOL
INSOL
GFP

S3 FigB

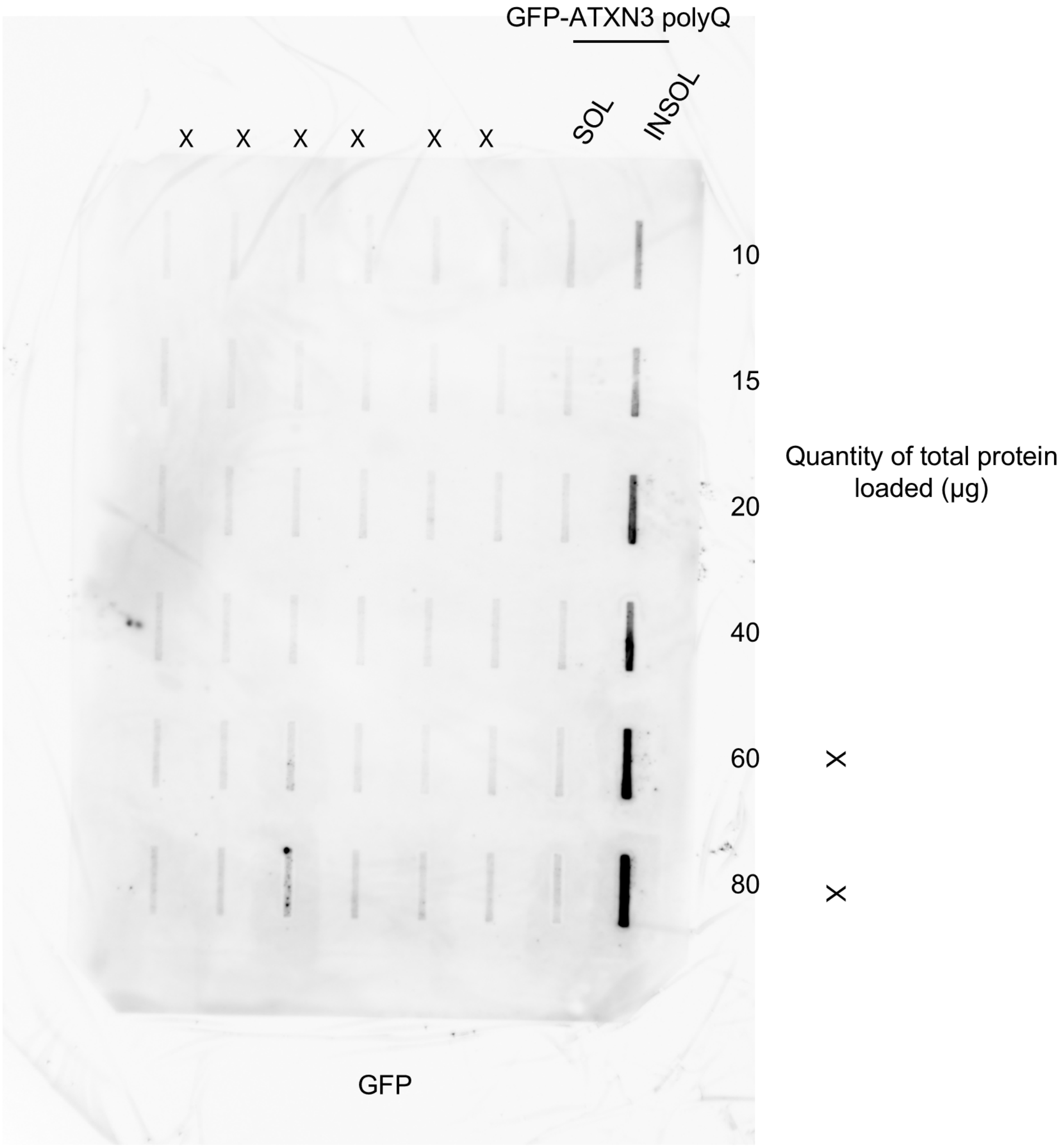
GFP-ATXN3 polyQ
X
X
X
X
X
X
SOL
INSOL
10
15
20
40
60
80
Quantity of total protein loaded (µg)
X
X
GFP

S4 Fig

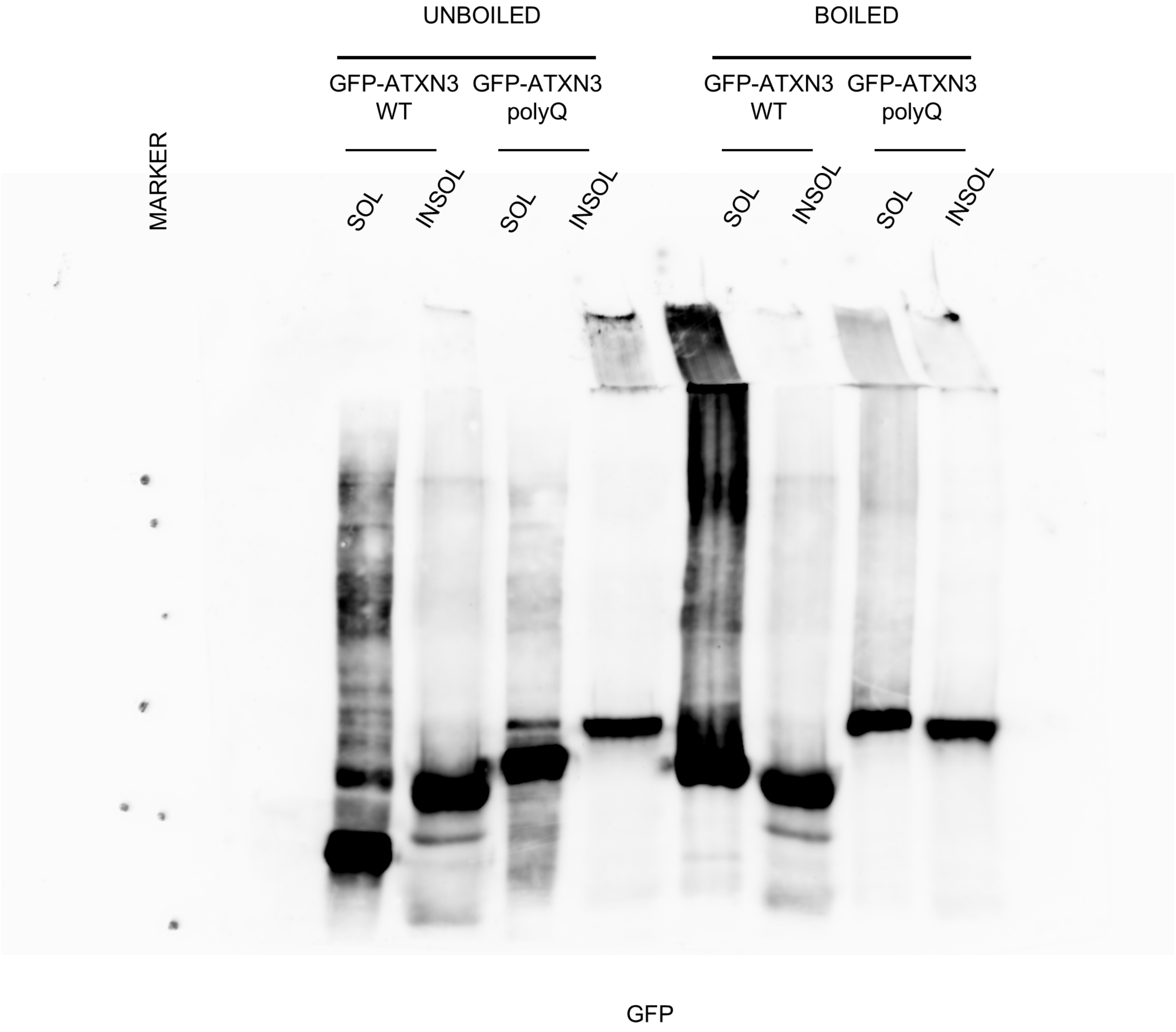
UNBOILED
BOILED
GFP-ATXN3 WT
GFP-ATXN3 polyQ
GFP-ATXN3 WT
GFP-ATXN3 polyQ
MARKER
SOL
INSOL
SOL
INSOL
SOL
INSOL
SOL
INSOL
GFP
