# Supplementary material for: Biochemical analysis to study wild-type and polyglutamine-expanded ATXN3 species
Source: PLoS One. 2024 Dec 23;19(12):e0315868. doi: 10.1371/journal.pone.0315868 (PMC11666052; doi:10.1371/journal.pone.0315868)
Supplement: Attachment — Submitted filename: Quinet et al response to reviewer.pdf [file pone.0315868.s008.pdf]

Journal Requirements:

Please review your reference list to ensure that it is complete and correct. If you have cited papers that have been retracted, please include the rationale for doing so in the manuscript text, or remove these references and replace them with relevant current references. Any changes to the reference list should be mentioned in the rebuttal letter that accompanies your revised manuscript. If you need to cite a retracted article, indicate the article's retracted status in the References list and also include a citation and full reference for the retraction notice.

Additional Editor Comments (if provided):

The manuscript is much improved. Please address the one comment by reviewer #2.

[Note: HTML markup is below. Please do not edit.]

Reviewers' comments:

Reviewer's Responses to Questions

Reviewers' comments:

Reviewer's Responses to Questions
Comments to the Author

Reviewers' comments:

Reviewer's Responses to Questions
Comments to the Author

1. Does the manuscript report a protocol which is of utility to the research community and adds value to the published literature?
Reviewer #1: Yes
Reviewer #2: Yes

---

2. Has the protocol been described in sufficient detail?

To answer this question, please click the link to protocols.io in the Materials and Methods section of the manuscript (if a link has been provided) or consult the step-by-step protocol in the Supporting Information files.

The step-by-step protocol should contain sufficient detail for another researcher to be able to reproduce all experiments and analyses.
Reviewer #1: Yes
Reviewer #2: Yes

---

3. Does the protocol describe a validated method?

The manuscript must demonstrate that the protocol achieves its intended purpose: either by containing appropriate validation data, or referencing at least one original research article in which the protocol was used to generate data.
Reviewer #1: Yes
Reviewer #2: Yes

---

4. If the manuscript contains new data, have the authors made this data fully available?

The PLOS Data policy requires authors to make all data underlying the findings described in their manuscript fully available without restriction, with rare exception (please refer to the Data Availability Statement in the manuscript PDF file). The data should be provided as part of the manuscript or its supporting information, or deposited to a public repository. For example, in addition to summary statistics, the data points behind means, medians and variance measures should be available. If there are restrictions on publicly sharing data—e.g. participant privacy or use of data from a third party—those must be specified.
Reviewer #1: Yes
Reviewer #2: N/A

---

5. Is the article presented in an intelligible fashion and written in standard English?

PLOS ONE does not copyedit accepted manuscripts, so the language in submitted articles must be clear, correct, and unambiguous. Any typographical or grammatical errors should be corrected at revision, so please highlight any specific errors that need correcting in the box below.
Reviewer #1: Yes
Reviewer #2: Yes

---

6. Review Comments to the Author

Please use the space provided to explain your answers to the questions above. You

may also include additional comments for the author, including concerns about dual publication, research ethics, or publication ethics. (Please upload your review as an attachment if it exceeds 20,000 characters)
Reviewer #1: I have answered all the questions above favorably. Thank you for carefully addressing reviewer comments.

We thank the reviewer for his/her comments to help to improve our manuscript.

Reviewer #2: Thank you for thoroughly addressing my previous comments. However, there is one point related to the new data that requires clarification. On page 9, line 236 the revised text states that "…two ATXN3 antibodies and the polyglutamine antibody recognized some proteins smaller than the GFP ATXN3 polyQ monomer, that were not recognized by the anti-GFP antibody (Fig 2A, S2 FigB). These bands could either correspond to overexpressed GFP-ATXN3 polyQ that was processed to smaller fragments without GFP or to (modified forms of) endogenous ATXN3."
It is unclear which smaller bands the authors are referring to in Figure S2B; I assume it is the double-band in the soluble fraction, but this should be specified. Additionally, it seems unlikely that the smaller band corresponds to endogenous ATXN3, given the cross-reactivity with the 1C2 antibody, which recognizes expanded polyglutamine. Aside from this, my concerns have been addressed.

Thanks to the reviewer for his/her comments. To address this last comment, we added new panels in supplemental figure 2 (S2 Fig C) in which we analysed total extracts of control and ATXN3-depleted cells together with the fractionation of GFP-ATXN3 polyQ expressing cells by western blot, using the two ATXN3 antibodies used in S2 Fig B. The double band that corresponds to endogenous ATXN3, was marked with an asterisk in the figure S2 for clarification. This double band migrated at the same mobility as bands present after fractionation of cells expressing GFP-ATXN3 polyQ. Therefore, we find it likely that these bands correspond to the endogenous ATXN3 protein. Accordingly, new text was added (lines 241-244), the S2 figure legend was adapted (lines 425-430) in the main manuscript and information about the siRNA oligonucleotides was included in the S1 file.

---

7. PLOS authors have the option to publish the peer review history of their article (what does this mean?). If published, this will include your full peer review and any attached files.

If you choose "no", your identity will remain anonymous but your review may still be made public.

Do you want your identity to be public for this peer review? For information about this choice, including consent withdrawal, please see our Privacy Policy.
Reviewer #1: No

Reviewer #2: No

[NOTE: If reviewer comments were submitted as an attachment file, they will be attached to this email and accessible via the submission site. Please log into your account, locate the manuscript record, and check for the action link "View Attachments". If this link does not appear, there are no attachment files.]

While revising your submission, please upload your figure files to the Preflight Analysis and Conversion Engine (PACE) digital diagnostic tool, https://pacev2.apexcovantage.com/. PACE helps ensure that figures meet PLOS requirements. To use PACE, you must first register as a user. Registration is free. Then, login and navigate to the UPLOAD tab, where you will find detailed instructions on how to use the tool. If you encounter any issues or have any questions when using PACE, please email PLOS at figures@plos.org. Please note that Supporting Information files do not need this step.
